# Attitude of Nutrition Experts Toward Psychotherapy and Virtual Reality as Part of Obesity Treatment—An Online Survey

**DOI:** 10.3389/fpsyt.2022.787832

**Published:** 2022-04-25

**Authors:** Kathrin Gemesi, Sophie Laura Holzmann, Regine Hochrein, Nina Döllinger, Carolin Wienrich, Natascha-Alexandra Weinberger, Claudia Luck-Sikorski, Christina Holzapfel

**Affiliations:** ^1^School of Medicine, Institute for Nutritional Medicine, Technical University of Munich, Munich, Germany; ^2^Research Group “Chronic Diseases and Psychological Health” (COPE), SRH, University of Applied Health Sciences, Gera, Germany; ^3^Human-Technology-Systems, University of Würzburg, Würzburg, Germany; ^4^Integrated Research and Treatment Center (IFB) AdiposityDiseases, Leipzig University – Medical Center, Leipzig, Germany

**Keywords:** body image therapy, weight loss, lifestyle intervention, obesity, virtual reality

## Abstract

**Background:**

The management of obesity requires lifestyle-based interventions covering nutrition, physical activity, and behavior. As part of cognitive behavioral therapy (CBT), body image therapy approaches can be used not only by psychotherapists. One tool to conduct behavioral therapy is virtual reality (VR). It is unknown, whether nutrition experts conduct behavioral therapy, and whether they would like to use VR technology as a tool to conduct body image therapy as part of obesity management.

**Objective:**

This survey aimed to collect data from nutrition experts treating people with obesity about the *status quo* regarding behavioral and body image therapy as part of obesity management, and regarding their attitude toward VR in obesity therapy.

**Methods:**

The survey was conducted online in autumn 2020. Participants were recruited digitally through expert and professional associations. The standardized questionnaire included items concerning sociodemographic, professional status, behavioral therapy, body image, and VR. The descriptive analysis was performed with Excel, the subgroup analyses with R.

**Results:**

Data from 158 nutrition experts was analyzed. Participants were mostly female (98/102, 96.1%) and had a mean age of 45.6 ± 11.3 years (*n* = 101). Most of the survey participants (93/124, 75.0%) stated to use behavioral treatment methods in case of weight reduction as the primary target. More than half of the participants stated to address body image (99/150, 66.0%). Almost all (111/112, 99.1%) nutrition experts have never used VR-glasses. The suitability and importance of VR technology as part of obesity therapy was estimated as neutral by around 50%. Overall, no statistically significant difference could be shown between age groups regarding attitudes toward VR in obesity treatment.

**Conclusion:**

The results of this non-representative survey indicate that nutrition experts do not use VR technology in nutrition counseling sessions to treat obesity. In addition, survey participants have a positive attitude to VR technology, whereas they are not familiar with this technology. In future, VR technology might support nutrition experts of every age using elements of body image therapy.

**Survey Registration:**

The German Register of Clinical Studies (Registration Number: DRKS00022853).

## Introduction

Overweight and obesity are major public health challenges affecting an increasing number of people worldwide (1, 2). National (e.g., German Obesity Association, DAG) and international societies (e.g., European obesity community, EASO) provide evidence-based guidelines including lifestyle-based interventions for the management of overweight and obesity in adults (3). These include the reduction of energy intake, the increase of physical activity, and the use of behavioral weight loss approaches like cognitive behavioral therapy (CBT) including elements like reinforcement, stimulus control, goal setting, and problem solving (4, 5).

Different tools are available to conduct CBT, yet traditional face-to-face settings are time- and cost-intensive (6, 7). Virtual reality (VR) technology is a tool that gained interest also for behavioral therapy. It has the ability to simulate a real-world setting in a controlled environment (8) that could support the integration of behavioral weight loss approaches in obesity management concepts. The Behavioral Framework of Immersive Technologies (BehaveFIT) describes how the potentials of VR can help overcome psychological barriers (9).

The VR cue exposure (VR-CE) and VR reference frame shifting (VR-RFS) are two VR-based cognitive behavioral approaches that are often used in the case of eating disorders, but also increasingly for obesity. While the VR-CE targets symptoms like food cravings and food-related anxiety to reduce overeating (10, 11), the VR-RFS targets negative memories of the body that are expected to possibly result in eating disorders or obesity to modify disturbances in body self-perception (11, 12).

Regarding obesity and binge eating disorders, Riva and colleagues showed that a short-term VR therapy is more effective than traditional CBT in reducing body dissatisfaction (13). Additionally to VR itself, avatars could be an opportunity to correct a negative body image, especially concerning disturbances in body weight perception (14–16). Avatars allow for a simulation of bodily changes that can help re-evaluate the own actual body weight and for evaluating the degree of body weight misperception of a person. Wolf and colleagues found out, that embodying a generic avatar can lead to different body weight estimations depending on the discrepancy between the own weight and the avatars weight (17, 18). Using VR technology might be limited by high costs (13), side effects (“simulation sickness”) (19), and missing virtual body ownership [“subjective experience to self-attribute a virtual body” (17)] and agency [“subjective experience of having control of a body” (17)] influencing the acceptance of the virtual body as the own body (20).

Randomized controlled trials (RCT) investigated the potential of VR-enhanced CBT for the management of obesity. The RCT by Manzoni et al. examined 163 women with morbid obesity for 6 months with a follow-up of 1 year (21). Participants were randomized to a standard behavioral program (SBP, including nutrition, medical, physical, and psychological therapy), or to SBP combined with standard CBT, or to SBP combined with VR-enhanced CBT. The VR-enhanced CBT was conducted in a virtual world that included 14 different virtual environments in which participants could deal with daily situations (e.g., home, supermarket) by using a personalized avatar (virtual 3D model). After 1 year of follow-up, more participants of the “VR-enhanced CBT” group had maintained or improved weight loss compared to the SBP group. Eating behavior and body satisfaction improved after the treatment but did not differ significantly between the three groups. The study was limited by a dropout rate of 28.5% and by exclusion of males. In another RCT 146 adults with overweight or obesity had received either access to the weight management program Weight Watchers (WW) alone or combined with web-based VR sessions using avatars (=virtual 3D representations of the body) (22). After 6 months, participants who had received the VR-enhanced intervention had shown significantly more weight loss compared to the control group, but no between-groups difference could be shown regarding self-reported adoption of behaviors. The study was limited by missing data.

The CBT approaches for people with obesity also address body image by using e.g., mirror exposure (23). In a systematic review and meta-analysis, it is described that people with obesity were unable to estimate their body size/shape correctly (=body image distortion) or they did not like or accept their body as it is (=body image dissatisfaction) (16). A meta-analysis by Weinberger et al. (24) could show that people with obesity were significantly more often affected by body dissatisfaction compared to persons with normal weight. According to Rosen et al. (25) weight loss as part of a program including behavioral approaches can improve body image, whereas weight regain can again lead to a negative body image. Results of a 16 week weight loss study and 1 year of follow-up including 158 healthy women with overweight and obesity indicate that body disturbance can be a predictor for stopping or failing a weight loss program (26). In conclusion, weight management in combination with body image therapy could help to achieve weight loss and maintenance, and develop and preserve at the same time a positive body image.

To investigate the public opinion a video presenting using VR in health care was distributed on the social media platform Facebook (27). Almost 75% of the Facebook users' comment included positive remarks about VR (27). Because VR-enhanced therapy often requires a clinician to support the patient, previous studies have examined the clinicians' attitude toward the use of VR technology in therapy. Lindner et al. (28) investigated the attitude toward and familiarity specifically with VR-CE therapy among cognitive behavior therapists (predominantly psychologists) by using a survey. The majority of participants stated to have no experience in using VR in clinical practice, and the familiarity with VR in general and VR-CE therapy resulted to be low. The attitude toward VR-CE as a tool to treat mental disorders was overall positive.

In summary, using VR technology has the potential to improve the CBT of obesity, which is up to now mostly dependent on face-to-face real world interactions. Previous studies showed an overall positive attitude of professionals in the field of mental health toward VR in therapy. According to the European Clinical Practice Guidelines, behavioral interventions are not reserved for psychotherapists but should also be conducted by other experts, such as nutrition experts, because lifestyle-based interventions for managing obesity should combine behavioral and nutritional therapy as well as physical activity (4).

The “Virtual Reality Therapy by Stimulation of Modulated Body Perception (ViTraS)” project aims to develop a new VR tool including an avatar addressing the body image of people with obesity (14). This target group-specific online survey aimed to address the following research questions in order to develop a new VR tool: (1) Do nutrition experts use behavioral and body image therapy in their obesity therapy? (2) What is the nutrition experts' attitude toward VR in obesity treatment and can they imagine to use this technology for obesity treatment? (3) Are there statistically significant differences between young and middle-aged nutrition experts regarding their attitudes toward VR technology?

## Methods

### Design

The Ethical Committees of the School of Medicine at the Technical University of Munich and the Friedrich-Schiller-University in Jena approved this open online survey (ethical vote: 410/20S, 2020-1885-Bef), which was performed in October 2020 throughout Germany. The survey invitation included a link guiding participants to the online survey on the platform SoSci Survey (V3.1.06). The recruitment was conducted mainly through expert associations (e.g., German Nutrition Society, DGE), professional associations (e.g., Professional Association of Oecotrophology, VDOE), and digital communication channels (e.g., newsletters, social media, and email). Included participants had to be of adult age, to have good German language skills, to be nutrition experts (practicing or studying/in training), and to treat people with overweight or obesity. Eligibility was checked by collecting this information by self-reporting in the beginning of the survey (e.g., “Do you treat patients with overweight/obesity?”).

Since the invitation was delivered electronically, the exact number of invitations and the response rate is unknown. All participants gave informed consent to participate.

Before answering the questions participants had to confirm the data privacy statement and that they are of legal age. No incentives were offered to the participants.

### Questionnaire

The 56-item questionnaire was developed by a multidisciplinary team of nutritionists, psychologists, and computer scientists. After pretesting, the questionnaire included (closed, open, single, or multiple choice) questions referring to the profession (13 questions), body image (7 questions), behavioral treatment techniques (3 questions), communication with patients (7 questions), VR (11 questions), perception exercises (10 questions), socio-demographics (2 questions), and feedback (3 fields). The survey started with an introduction and some information about data privacy and protection. Neutral answer options like “occasional,” “other,” or “neutral” were provided if indicated. This work focuses on a selection of 23 questions (sociodemographic, professional status, use of behavioral and body image therapy, attitude toward VR), which are named in the respective table legends, to address the research questions. Questions not presented were focused on psychological and technical issues for the design and development of the VR environment within the ViTraS project.

### Statistical Analyses

Integrity and plausibility were checked. Descriptive data analyses (frequencies, percentages, standard deviation, and mean) were performed using Excel 2016 (Microsoft Corp). Since participants were able to quit the survey at any time and some data had to be excluded because of inconsistent answers, sample size differs between questionnaire items. The sample size was appropriate to perform age group specific analyses using R (V4.1.0). Two age groups 18 to 39 years (young) and 40 to 64 years (middle-aged) were defined. The relationship between age groups and VR-related results of the survey was assessed using Fisher's exact test. In advance, normality of age was tested by performing Shapiro-Wilk test and variance homogeneity was checked by using Levene test. No gender specific analyses were performed (96.1% women). *P*-values < 0.05 were considered as statistically significant.

## Results

### Characteristics

Participants (N_max_ = 158) were 45.6 ± 11.3 years old. Most of the participants were aged 40 to 64 years (68/101, 67.3%), female (98/102, 96.1%), and stated to use predominantly individual counseling (147/158, 93.0%) for patients with overweight/obesity (Table 1).

**Table 1:** Characteristics of the survey population.

				***n* (%)**
**Age in years (*****N*** **=** **101)**				
18–39				33 (32.7)
40–64				68 (67.3)
**Sex (*****N*** **=** **102)**				
Female				98 (96.1)
Male				4 (3.9)
		**Rarely/never**	**Occasional**	**Often/very often**
		***n*** **(%)**	***n*** **(%)**	***n*** **(%)**
**Treatment setting** ^**A**^				
Individual counseling/ therapy (*N* = 158)		2 (1.3)	9 (5.7)	147 (93.0)
Group counseling/ therapy (*N* = 158)		73 (46.2)	25 (15.8)	60 (38.0)
Other (*N* = 157)		127 (80.9)	19 (12.1)	11 (7.0)

^a^ *How often do you use the following forms of treatment for your patients with overweight/obesity?*

### Behavioral Treatment Approaches

The use of behavioral treatment approaches for patients with overweight/obesity is shown in Table 2. The majority of participants claimed to often use treatment approaches like promotion of motivation (137/151, 90.7%), target agreements (129/151, 85.4%), and relapse prevention (96/151, 63.6%) but never or rarely exposition by video recording (142/151, 94.0%) or mirror image (127/151, 84.1%). Three-quarters of participants (93/124, 75.0%) stated to use behavioral treatment methods in case of weight reduction as the primary target (**Table 4**). Furthermore, most of the participants responded to never or rarely use the material and symbolic reinforcers like money (82/88, 93.2%), token (74/88, 84.1%), stamps (66/88, 75.0%), or diligence points (59/88, 67.0%) (Table 2). In contrast, the majority of participants stated to often use social reinforcers like interest (84/88, 95.5%), praise (82/88, 93.2%), or activities (47/88, 53.4%).

**Table 2:** Behavioral and body image therapy approaches for patients with overweight/obesity.

	**Rarely/ never**	**Occasional**	**Often/ very often**
	***n* (%)**	***n* (%)**	***n* (%)**
**Behavioral therapy**			
**Treatment approaches**^**A**^ **(*****N*** **=** **151)**			
Target agreements	6 (4.0)	16 (10.6)	129 (85.4)
Training of problem-/conflict-solving	26 (17.2)	34 (22.5)	91 (60.3)
Reinforcers strategy	29 (19.2)	42 (27.8)	80 (53.0)
Promotion of motivation	4 (2.6)	10 (6.6)	137 (90.7)
Stimulus control	54 (35.8)	43 (28.5)	54 (35.8)
Relapse prevention	21 (13.9)	34 (22.5)	96 (63.6)
Cognitive restructuring	47 (31.1)	32 (21.2)	72 (47.7)
Stress management	25 (16.6)	46 (30.5)	80 (53.0)
Mindfulness training	29 (19.2)	34 (22.5)	88(58.3)
Exposition by pleasure training	57 (37.7)	42 (27.8)	52 (34.4)
Exposition by food	53 (35.1)	46 (30.5)	52 (34.4)
Exposition by video recording	142 (94.0)	6 (4.0)	3 (2.0)
Exposition by mirror image	127 (84.1)	17 (11.3)	7 (4.6)
Social competence training	81 (53.6)	39 (25.8)	31 (20.5)
Confrontation training	121 (80.1)	23 (15.2)	7 (4.6)
Other	103 (68.2)	32 (21.2)	16 (10.6)
**Material/symbolic reinforcers** ^**B**^			
Token (e.g., coins) (*N* = 85)	74 (84.1)	6 (6.8)	5 (5.7)
Pictures (*N* = 84)	33 (37.5)	20 (22.7)	31 (35.2)
Diligence points (*N* = 84)	59 (67.0)	17 (19.3)	8 (9.1)
Stamps (*N* = 85)	66 (75.0)	12 (13.6)	7 (8.0)
Money (*N* = 85)	82 (93.2)	3 (3.4)	0
**Social reinforcers** ^**C**^			
Praise (*N* = 88)	2 (2.3)	4 (4.5)	82 (93.2)
Applause (*N* = 86)	46 (52.3)	18 (20.5)	22 (25.0)
Interest (*N* = 88)	1 (1.1)	3 (3.4)	84 (95.5)
Activities (*N* = 87)	20 (22.7)	20 (22.7)	47 (53.4)
Other (*N* = 52)	39 (44.3)	4 (4.5)	9 (10.2)
**Body image therapy**			
**Treatment approaches**^**D**^ **(*****N*** **=** **93)**			
Imagination exercises	55 (59.1)	23 (24.7)	15 (16.1)
Palpation/drawing exercises	76 (81.7)	13 (14.0)	4 (4.3)
Modeling exercises	88 (94.6)	3 (3.2)	2 (2.2)
Mirror confrontation	78 (83.9)	11 (11.8)	4 (4.3)
Video confrontation	92 (99.0)	1 (1.1)	0
Confrontation with everyday situations	33 (35.5)	22 (23.7)	38 (40.9)
Other	56 (60.2)	21 (22.6)	16 (17.2)
**Registration methods**^**E**^ **(*****N*** **=** **73)**			
Drawing/painting persons	64 (87.7)	6 (8.2)	3 (4.1)
Drawing body silhouette	54 (74.0)	12 (16.4)	7 (9.6)
Video recordings	72 (98.6)	1 (1.4)	0
Mirror	61 (83.6)	9 (12.3)	3 (4.1)
Distorting mirror	73 (100.0)	0	0
Picture assigning	59 (80.8)	9 (12.3)	5 (6.8)
Standardized questionnaires	47 (64.4)	18 (24.7)	8 (11.0)
Other	50 (68.5)	12 (16.4)	11 (15.1)

^a^ *How often do you use the following treatment approaches in your patients with overweight/obesity?*

*^b^Which material or symbolic reinforcers do you use how often in the therapy of overweight/obesity?, Other not shown*.

*^c^Which social reinforcers do you use how often in the therapy of overweight/obesity?*

*^d^How often do you use the following treatment approaches regarding body image therapy in patients with overweight/obesity?*

*^e^How often do you use the following registration methods on the subject of “body image” in body image therapy in patients with overweight/obesity?*

### Body Image Therapy

Regarding body image therapy, most of the participants (99/150, 66.0%) stated to address the topic “body image” with their patients with overweight/obesity. The majority of them (86/99, 86.9%) do not use manuals (**Table 4**). Table 2 shows the frequency of the use of treatment approaches and registration methods. The majority of participants stated to rarely or never use video confrontation (92/93, 99.0%), modeling exercise (88/93, 94.6%), and mirror confrontation (78/93, 83.9%). Similarly, most of the participants claimed to never or rarely use the queried registration methods including e.g., distorting mirror (73/73, 100.0%), video recordings (72/73, 98.6%), drawing/painting persons (64/73, 87.7%), and mirror (61/73, 83.6%).

### Virtual Reality

The answers given regarding the suitability of VR glasses and the importance of VR techniques in the therapy of patients with overweight/obesity are shown in Table 3. The application of VR glasses as part of an individual or group therapy was considered neither unsuitable nor suitable by nearly half of the participants (individual: 52/106, 49.1%; group: 50/106, 47.2%). The suitability of two different functions of self-touch, an exercise in VR to enhance body awareness, was mainly assessed as neutral (function 1: 51/102, 50.0%, function 2: 58/102, 56.9%). Asking the participants about the importance of VR in the therapy at the moment and in the future, 46.1% (47/102), and 56.2% (59/105) responded neutrally. No statistically significant difference between age groups could be shown regarding the stated suitability of VR glasses and the importance of VR techniques in the therapy of patients with overweight or obesity (all *p* > 0.05).

**Table 3:** Suitability/importance of virtual reality (VR) in the therapy of overweight/obesity.

	**Unsuitable/very unsuitable**			**Neutral**			**Suitable/very suitable**			***p*-value**
	**Total**	**18–39 y**	**40–64 y**	**Total**	**18–39 y**	**40–64 y**	**Total**	**18–39 y**	**40–64 y**	
	***n* (%)**	***n* (%)**	***n* (%)**	***n* (%)**	***n* (%)**	***n* (%)**	***n* (%)**	***n* (%)**	***n* (%)**	
**Suitability**										
Individual therapy (*N* = 106)^a^	18 (17.0)	5 (15.2)	10 (14.7)	52 (49.1)	18 (54.5)	32 (47.1)	36 (34.0)	10 (30.3)	26 (38.2)	0.70
Group therapy (*N* = 106)^b^	40 (37.7)	10 (30.3)	29 (42.6)	50 (47.2)	19 (57.6)	28 (41.2)	16 (15.1)	4 (12.1)	11 (16.2)	0.36
Function 1: self-touch results in uncovering the body (*N* = 102)^c^	23 (22.5)	7 (21.2)	15 (22.1)	51 (50.0)	15 (45.5)	36 (52.9)	28 (27.5)	11 (33.3)	17 (25.0)	0.12
Function 2: self-touch results in painting the body (*N* = 102)^d^	21 (20.6)	4 (12.1)	16 (23.5)	58 (56.9)	20 (60.6)	38 (55.9)	23 (22.5)	9 (27.3)	14 (20.6)	0.47
	**Less important/not important**			**Neutral**			**Important/very important**			***p*** **-value**
	**Total**	**18–39 y**	**40–64 y**	**Total**	**18–39 y**	**40–64 y**	**Total**	**18–39 y**	**40–64 y**	
	***n*** **(%)**	***n*** **(%)**	***n*** **(%)**	***n*** **(%)**	***n*** **(%)**	***n*** **(%)**	***n*** **(%)**	***n*** **(%)**	***n*** **(%)**	
**Importance**										
At the moment (*N* = 102)^e^	25 (24.5)	10 (30.3)	14 (20.6)	47 (46.1)	16 (48.5)	31 (45.6)	30 (29.4)	7 (21.2)	23 (33.8)	0.12
In future (*N* = 105)^f^	23 (21.9)	7 (21.2)	14 (20.6)	59 (56.2)	18 (54.5)	39 (57.4)	23 (21.9)	8 (24.2)	15 (22.1)	0.44

*y, years*.

*^a^What applies to you? Complete the sentence “The application of virtual reality-glasses as part of an individual therapy I consider to be …”*.

*^b^What applies to you? Complete the sentence “The application of virtual reality-glasses as part of a group therapy I consider to be …”*.

*^c^The virtual reality exercise “self-touch” results in uncovering the respective body site. How would you assess the suitability of this exercise?*

*^d^The virtual reality exercise “self-touch results in painting the respective body site. How would you assess the suitability of this exercise?*

*^e^How important do you assess the technic of virtual reality in the therapy of patients with overweight/obesity?*

*^f^In your opinion, how important will virtual reality be in the therapy of patients with overweight/obesity in the future?*

In Table 4, results on the subject of body image and behavioral therapy, communication, and VR are shown. Regarding body image therapy, both age groups stated equally to address the topic of “body image” with their patients with overweight/obesity but the use of manuals was shown to be statistically significantly different between the two age groups (*p* = 0.01). Most of the nutrition experts (90/117, 76.9%) stated to agree on the transmission of facial expression and gestures through digital media, with a statistically significant difference between age groups (*p* = 0.04). In addition, the majority of them responded that communicating with their patients in person is important to them (116/118, 98.3%) (data not shown). Furthermore, more than half (57/106, 53.8%) of the participants see VR therapy as a chance for patients with difficulties in access public transport when coming to therapy. However, most of the nutrition experts (59/106, 55.7%) stated that their patients are not affected by difficulties in access public transport. The majority of experts never used VR glasses before (111/112, 99.1%) and would not be willing to buy VR glasses (75/105, 71.4%). Nevertheless, most of the nutrition experts (88/102, 86.3%) would be willing to do nutrition counseling in a virtual supermarket.

**Table 4:** Body image and behavioral therapy, communication, and virtual reality.

	**Yes**			**No**			***p*-value**
	**Total**	**18–39 y**	**40–64 y**	**Total**	**18–39 y**	**40–64 y**	
	***n* (%)**	***n* (%)**	***n* (%)**	***n* (%)**	***n* (%)**	***n* (%)**	
**Body image therapy**							
Addressing body image (*N* = 150)^a^	99 (66.0)	16 (48.5)	46 (67.6)	51 (34.0)	17 (51.5)	22 (32.4)	0.08
Using manuals (*N* = 99)^b^	13 (13.1)	5 (15.2)	2 (2.9)	86 (86.9)	11 (33.3)	44 (64.7)	0.01
**Behavioral therapy (*****N*** **=** **124)**							
Use in case of the primary target of weight reduction^c^	93 (75.0)	26 (78.8)	52 (76.5)	31 (25.0)	7 (21.2)	16 (23.5)	1
**Communication (*****N*** **=** **117)**							
Transmission of facial expression and gestures^d^	90 (76.9)	30 (90.9)	49 (72.1)	27 (23.1)	3 (9.1)	19 (27.9)	0.04
**Virtual reality (VR)**							
VR-therapy is a chance for patients with difficulties in access public transport (*N* = 106)^e^	57 (53.8)	21 (63.6)	34 (50.0)	49 (46.2)	12 (36.4)	34 (50.0)	0.21
My patients have difficulties in access public transport (*N* = 106)^f^	47(44.3)	19 (57.6)	27 (39.7)	59 (55.7)	14 (42.4)	41 (60.3)	0.14
Already used VR-glasses (*N* = 112)^g^	1 (0.9)	0	0	111 (99.1)	33 (100)	68 (100)	-
Willing to buy VR-glasses (*N* = 105)^h^	30 (28.6)	9 (27.3)	21 (30.9)	75 (71.4)	24 (72.7)	47 (69.1)	0.82
Willing to do nutrition counseling in a virtual supermarket (*N* = 102)^i^	88 (86.3)	30 (90.9)	58 (85.3)	14 (13.7)	3 (9.1)	10 (14.7)	0.54

*y, years*.

*^a^Do you address the topic “body image” in your patients with overweight/obesity?*

*^b^Do you use manuals for body image therapy in your patients with overweight/obesity?, Only participants who stated to address body image could answer this question.*

*^c^Do you use behavioral treatment methods in case of the primary target of weight reduction?*

*^d^Would you agree with the transmission of facial expression and gestures through digital media (e.g., video) during the therapy of overweight/obesity?*

*^e^Do you see virtual reality as an opportunity for your patients with overweight/obesity with difficulties in access public transport?*

*^f^Are your patients affected by difficulties in access public transport?*

*^g^Have you already used virtual reality-glasses in a meeting with your patients with overweight/obesity?*

*^h^Would you be willing to buy virtual reality-glasses for the application in your therapy?*

*^i^Can you imagine doing nutrition counseling in a virtual supermarket (=virtual nutrition counseling)?*

## Discussion

This survey has shown that most of the participants provide individual counseling for patients with overweight/obesity and that the survey population uses classic behavioral therapy approaches, but fewer body image therapy approaches. Regarding VR technology, the participants had no experience yet. The suitability and importance of VR in the treatment of overweight/obesity were estimated as neutral. The attitude toward the use of VR in obesity therapy was not different between age groups.

Individual therapy is the usual setting that is used by nutrition experts for obesity treatment. This might be explained by the fact that obesity is complex and multifactorial (6, 7) that consequently acquires an individual therapeutic approach. Furthermore, patients often experience social exclusion that was shown to result in an increased feeling of shame (29). Face-to-face nutrition counseling might increase self-confidence and decrease the feeling of social exclusion. More than half of the participants of this online survey were women. This finding matches with experience showing that the majority of nutrition experts is female.

Survey participants stated to use basic behavioral therapy approaches but not others like mirror exposure. A similar result has been observed for social reinforcers that are popular tools, but not for material/symbolic reinforcers. These results indicate that nutrition experts try to use behavior change techniques during nutrition counseling sessions, but are not familiar with them. This is in line with the European Clinical Practice Guidelines for the management of obesity in adults which intends not only psychotherapists but other health professionals such as dieticians to provide behavioral therapy (4). Traditionally, dieticians have not been trained in behavioral treatment techniques (30). A survey showed that dietitians feel to have a gap in their behavioral change skills (31). The same applies to the result that survey participants stated to address body image. This fact indicates a significant drawback in obesity therapy. Psychotherapists are trained in behavioral and body image therapy approaches but do not treat people living with overweight or obesity with the primary aim of weight loss. Consequently, nutritionists, who are the primary contact persons for people with overweight and obesity, are forced to provide behavioral change techniques without having advanced knowledge to offer an integral obesity treatment.

At this point, VR technology could be an opportunity to close a gap by facilitating nutrition experts to use elements of behavioral and body image therapy within a nutrition-based context. Furthermore, VR could be a tool for people with overweight or obesity to practice self-help in addition to professional therapy. In addition, VR technology can facilitate personalized CBT for obesity, which might be more effective. A recent review by Dalle Grave et al. describes the new approach of personalized CBT for obesity (CBT-OB) (32). It connects standard behavioral therapy for obesity (e.g., goal-setting, stimulus control, problem solving) with personalized cognitive strategies. According to the review, CBT-OB could be more effective than traditional lifestyle-changing programs for weight loss.

Nearly half of the survey participants assessed the suitability of VR technology for individual (49.1%) and group therapy (47.2%) neutral, just as the importance of this technology (at the moment: 46.1%, in future: 56.2%). On the one hand, this result suggests that nutrition experts are not averse to implement VR techniques into their counseling sessions. On the other hand, the survey participants had never been confronted with VR tools and therefore might not have been able to estimate it other than neutral. In contrast, 86.3% of the participants can imagine doing virtual nutrition counseling. The willingness of the survey participants to use a virtual supermarket as a setting for nutrition counseling can be explained by the fact that the supermarket is often regarded as a critical situation for people with overweight/obesity since a link between food offer, food choice, and body weight is suspected (33, 34). Consequently, the setting of a virtual supermarket is more familiar to nutrition experts and therefore easier to imagine and estimate.

The attitude toward using VR technology for obesity treatment was neutral. In contrast, Schwartzman et al. (35) who asked 262 psychotherapists (currently not using VR in their therapies) about their interest, knowledge, and perceptions regarding VR showed that participants seemed to have not too much interest in VR in clinical practice because of possible costs and need for extra training. Moreover, participants were not familiar with the benefits and applications of VR in treatment. In the study by Chung et al. (36) clinicians (primarily mental health nurses) and service managers were confronted with a VR scenario for obsessive-compulsive disorder treatment and interviewed about chances and possible barriers of VR technology. The overall opinion was that VR in mental health care could support the quality of health care and enhance patient engagement. However, participants were concerned about feasibility, resourcing, limitations, and safety of VR.

Although there are many promising approaches in VR research and although clinicians do not seem to be averse to VR in therapy, the transfer into practice is not very advanced. Therefore, more RCTs with people affected by obesity should be conducted. Literature, as well as RCTs using VR techniques for the treatment of overweight and obesity, are scarce, compared to e.g., eating disorders. The efficacy of VR-enhanced CBT in the treatment of binge-purging-type eating disorders (bulimia nervosa and binge eating disorder) compared to CBT, the usual first-line treatment in the management of eating disorders (37), was summarized in the meta-analysis by Low et al. (38). For the analysis, six RCTs with 297 participants in total were included. VR-enhanced CBT included elements like self-monitoring, coping strategies, confrontation with daily situations, relapse prevention, problem solving, and body image. Participants who underwent the VR-enhanced CBT showed a significantly larger decrease in binge frequency but did not differ in their overall body satisfaction from the CBT-group (38).

The potential of VR technology to enhance traditional obesity therapy is intensively investigated. Willem et al. (39) could show that people with obesity reported more often to have difficulties with emotion regulation compared to people with normal weight. This deficit seems to play a role in the development and maintenance of obesity (40). In a RCT conducted by Manzoni et al. (41) the intervention with VR-enhanced relaxation training about 3 weeks could reduce emotional eating in women with obesity more than traditional relaxation training and standard hospital-based care.

Although this target group-specific non-representative survey used a standardized questionnaire developed by a multidisciplinary team, the present survey is limited by the fact, that no data about the training of behavioral change techniques of nutritionists have been queried. Furthermore, no data of psychotherapists, who usually do behavioral and body image therapy, were collected. In addition, it has to be mentioned that knowledge of VR and familiarity therewith were not measured by the survey. Nutrition experts were only asked whether they already used VR-glasses in a meeting with their patients with overweight or obesity.

In relation to our research questions, the following statements can be made based on the results of this survey: (1) Nutrition experts conduct behavioral therapy but less body image therapy, whereas they use some elements of body image therapy. (2) The suitability and importance of VR in the treatment of overweight and obesity is estimated neutral. (3) No statistical significant differences between the two defined age groups could be shown. In our opinion, the VR technology could be used as an easily accessible tool for nutrition experts of every age conducting behavioral and body image therapy in the context of nutrition. Nutrition experts additionally trained in basics of body image and behavioral change techniques might provide better support to persons with overweight or obesity.

## Data Availability Statement

The raw data supporting the conclusions of this article will be made available by the authors, without undue reservation.

## Ethics Statement

The studies involving human participants were reviewed and approved by Ethical Committees of the School of Medicine at the Technical University of Munich and the Friedrich-Schiller-University in Jena (ethical vote: 410/20S, 2020-1885-Bef). The patients/participants provided their written informed consent to participate in this study.

## Author Contributions

SH, RH, and ND prepared the survey and collected the data. KG and CH performed data cleaning, statistical analyses, data interpretation, and manuscript preparation. SH, RH, ND, CW, N-AW, and CL-S carried out manuscript proof-reading. All authors contributed to the article and approved the submitted version.

## Funding

This article was written within the Virtual Reality Therapy by Stimulation of Modulated Body Perception (ViTraS) research consortium, financially supported by the German Federal Ministry of Education and Research (Bundesministerium für Bildung und Forschung, BMBF; grant numbers: 16SV8219, 16SV8221, and 16SV8224).

## Conflict of Interest

CH is a member of the scientific advisory board of the 4sigma GmbH (Oberhaching, Germany). The remaining authors declare that the research was conducted in the absence of any commercial or financial relationships that could be construed as a potential conflict of interest.

## Publisher's Note

All claims expressed in this article are solely those of the authors and do not necessarily represent those of their affiliated organizations, or those of the publisher, the editors and the reviewers. Any product that may be evaluated in this article, or claim that may be made by its manufacturer, is not guaranteed or endorsed by the publisher.
